# Lithium-induced neuroprotection is associated with epigenetic modification of specific BDNF gene promoter and altered expression of apoptotic-regulatory proteins

**DOI:** 10.3389/fnins.2014.00457

**Published:** 2015-01-14

**Authors:** Tushar Dwivedi, Hui Zhang

**Affiliations:** Department of Psychiatry, University of Illinois at ChicagoChicago, IL, USA

**Keywords:** lithium, bipolar, BDNF, apoptotic regulatory genes, methylation, hippocampal culture

## Abstract

Bipolar disorder (BD), one of the most debilitating mental disorders, is associated with increased morbidity and mortality. Lithium is the first line of treatment option for BD and is often used for maintenance therapy. Recently, the neuroprotective action of lithium has gained tremendous attention, given that BD is associated with structural and functional abnormalities of the brain. However, the precise molecular mechanism by which lithium exerts its neuroprotective action is not clearly understood. In hippocampal neurons, the effects of lithium (1 and 2 mM) on neuronal viability against glutamate-induced cytotoxicity, dendritic length and number, and expression and methylation of BDNF promoter exons and expression of apoptotic regulatory genes were studied. In rat hippocampal neurons, lithium not only increased dendritic length and number, but also neuronal viability against glutamate-induced cytotoxicity. While lithium increased the expression of BDNF as well as genes associated with neuroprotection such as Bcl2 and Bcl-XL, it decreased the expression of pro-apoptotic genes Bax, Bad, and caspases 3. Interestingly, lithium activated transcription of specific exon IV to induce BDNF gene expression. This was accompanied by hypomethylation of BDNF exon IV promoter. This study delineates mechanisms by which lithium mediates its effects in protecting neurons.

## Introduction

Bipolar disorder (BD) is one of the most debilitating disorders, which affects about 1% of the population in the world (Schroeder et al., 2011). Among various mental disorders, BD is associated with the highest risk of suicide (Nock et al., 2009), characterized by often cyclothymic and irritable temperaments, which leads to increased risk of suicide up to 20 times that of the average population (Pompili et al., 2013).

Several clinical studies suggest that BD is associated with changes in the brain structure and imbalance in signal transduction mechanisms that can lead to altered cellular integrity and synaptic plasticity. For example, a magnetic resonance spectroscopy (MRS) study demonstrates that BD is associated with neuronal and glial stress, neuronal atrophy, and cell death (Glitz et al., 2002). Further, bipolar patients show reduced gray matter volume in medial prefrontal cortex, amygdala, and ventral and orbitofrontal cortices (Brambilla et al., 2005). In addition, reduction in neuronal size and changes in neuronal density in medial prefrontal cortex of bipolar patients have been reported (Savitz et al., 2014). MRS studies also demonstrate that cellular signaling pathways that are critical for neuronal protection are significantly altered in prefrontal cortex, anterior cingulate cortex, and hippocampus of bipolar patients (Yildiz-Yesiloglu and Ankerst, 2006; Visnjic and Banfic, 2007).

Several studies suggest that brain-derived neurotrophic factor (BDNF), a protein involved in neuronal survival, dendritic branching, and synaptic plasticity (Huang and Reichardt, 2001), is lower in the brain and serum of bipolar patients (Cunha et al., 2006; Monteleone et al., 2008). Lower *BDNF* gene expression in bipolar patients could be associated with epigenetic modifications (D'Addario et al., 2012; Gao et al., 2012). BDNF mediates its neurotrophic action after binding to and activating tropomycin-receptor kinase B (TrkB). This leads to signaling cascade of events within neurons, mediated by extracellular signal-regulated kinases (ERKs) and phosphoinositide 3-kinase (PI3K) (Huang and Reichardt, 2001). Interestingly, it has been shown that not only is the activation of TrkB less active in the brain of bipolar patients but ablation of ERK activation can leads to hyperactivity in rodents (Engel et al., 2009). In addition, an association between *AKT1* genetic variants and BD has been shown which increases the suicidal risk in these patients (Karege et al., 2010; Magno et al., 2010). One of the major functions of BDNF-mediated ERK1/2 and PI 3-K/Akt signaling is to regulate proteins involved in cell survival. This occurs via the Bcl-2 family of proteins and the cysteine protease caspase family. Bcl2 family proteins are divided into two groups: anti-apoptotic (Bcl2 and Bclx_L_) and pro-apoptotic (Bax and Bak, Bid, Bad, and Bim) (Lucken-Ardjomande and Martinou, 2005). Pro-death Bcl2 family members initiate a cell death program, which involves opening of the mitochondrial permeability pore, decreases in the mitochondrial membrane potential, and the release of cytochrome C. This results in the nucleation of apoptosome, comprised of Apaf-1, cytochrome c, and caspase 9, which then cleaves and activates caspase-3, the main executioner of the apoptotic cascade (Yuan and Yankner, 2000). Bclx_L_ binds to Apaf-1 and blocks the ability of Apaf-1 to activate pro-caspase 9 which is antagonized by Bax (Schulze-Osthoff et al., 1998). The Bcl2 gene contains a CRE-binding site and activation of CREB via ERK1/2 can lead to increased synthesis of Bcl2 gene (Wilson et al., 1996). Expression of Bcl-x_L_ is regulated by p90RSK in response to ERK1/2 activation. A recent study shows polymorphism in Bcl-2 gene (rs956572) in BD patients (Soeiro-de-Souza et al., 2013). In addition, decreased density of neurons and glia and decreased size of neurons in frontal and subcortical areas of bipolar brain could be due to increased apoptosis (Gigante et al., 2011).

Lithium is the most effective mood stabilizer and is the first line of treatment for BD (Rybakowski, 2011). Though lithium is a highly prescribed medicine, its mechanism of action is not clear. Several lines of studies suggest that clinical efficacy of lithium is related to its effects on neuroprotection. This is evident from studies showing increased total gray matter content (Moore et al., 2000a) and enhanced levels of *N*-acetyl-aspartate, a marker of neuronal viability, by lithium in the brain of bipolar patients (Moore et al., 2000b). Bipolar patients, who are exposed to lithium, also have greater amygdala gray volume (Chang et al., 2005). Recently, Gildengers et al. (2014) reported that longer duration of lithium treatment is associated with higher white matter integrity. Similarly, the loss of the subgenual prefrontal cortex volume found in bipolar patients is suppressed in patients receiving lithium (Drevets, 2001). Given that lithium's neuroprotective action could be associated with clinical improvement, it is imperative to examine how lithium acts to protect neurons.

The present study aims to delineate such mechanisms. It is hypothesized that (1) lithium will protect neurons by increasing neuronal viability, dendritic length, and dendritic number, (2) lithium will increase the expression of *BDNF* gene and differentially regulate the expression of pro- and anti-apoptotic proteins, and (3) the effect of lithium on BDNF will be exon specific and will be epigenetically regulated.

## Materials and methods

### Neuronal culture and lithium treatment

Cryopreserved rat hippocampal neurons were obtained from UIC Research Resource Center (Chicago, IL) as a SPOT culture kit (provided by Dr. Lech Kiedrowaski). Neurons were suspended in neurobasal medium (Invitrogen, CA). The medium was supplemented with 1 mM glutamine and 1% B27 (Invitrogen, CA) and seeded onto culture adhesive coated surfaces provided with the Spot kit and kept in CO_2_ chamber at 37°C. After 3 days, 1 mM glutamine was replaced by 1 mM glutamax (Invitrogen, CA) in the culture medium. Seven days later, lithium chloride (Sigma, MO) was added to the culture medium to give the final concentration of 1 or 2 mM. Another group of neurons was treated with normal saline (0.9% NaCl_2_ in phosphate buffer saline). Neuronal cells were pretreated with lithium or normal saline for 36 h prior to the addition of glutamate (100 μM) for 12 h. Neuronal viability and morphology were studied. A separate set of neurons were treated with lithium (1 or 2 mM) for 48 h and the expression of BDNF as well as pro and anti-apoptotic genes was examined.

### Neuronal viability

Neuronal viability was assessed using MTT (3-(4,5-dimethylthiazol-2-yl)-2,5-diphenyltetrazolium bromide) reduction assay kit (Sigma, MO). MTT (0.5 mg/ml) was added to the culture medium (at a ratio of 1:10) after 48 h of lithium treatment. After 2 h incubation, the medium was taken out; formazon product was dissolved in dimethylsulfoxide; absorbance was measured at 570 nm and neuronal viability was calculated. The results are expressed as a percentage of viability of the control culture.

### Immunofluorescence staining of cultured neurons

To determine the effect of lithium on neuronal morphology, neuron cultures were fixed with 4% paraformaldehyde for 30 min at room temperature, permeabilized with 0.25% Triton X-100 in phosphate buffer saline for 10 min, blocked with 5% donkey serum (Jackson Laboratories, PA) for 1 h at room temperature, and then incubated for 16 h at 4°C with rabbit anti-microtubule-associated protein (MAP)2 antibody (Millipore, MA). Dilution of antibody was 1:1000. This was followed by incubation with fluorescein labeled donkey anti-rabbit IgG (1:200) (Jackson Laboratories, PA) for 1 h at room temperature. After washing in phosphate buffer saline, coverslips were inverted in slides over a drop of vectashield mounting medium for fluorescence with 4′,6-Diamidino-2-phenylindole (DAPI) (Vector Labs, CA). Images were obtained at 40× magnification using the camera attached to the microscope.

### Assays for dendritic outgrowth and neuroprotective activity

Ten photographs per well were obtained from three wells for each group. Thirty well-stained neurons that made connections to no more than two cells were selected for measurements of their primary dendritic length and dendritic number. Dendrites were defined as processes with a length longer than the cell diameter. Dendritic length was measured by the software from Neurolucida (MBF Bioscience, VT), whereas the percentage of cells forming dendrites was calculated.

### Determination of mRNA expression of BDNF, Bcl2, Bcl-_XL_, Bad, Bax, Caspase 3 by real time-reverse transcriptase polymerase chain reaction (RT-PCR)

RNA from hippocampal neurons were isolated using Trizol kit (Life Technologies, CA). The RNA yield and the ratio of absorbance at 260 to 280 nm (A_260_/A_280_ratio) were measured with the NanoDrop® Spectrophotometer (Thermo Scientific, DE). mRNA levels of various genes were determined using a two-step qPCR (Stratagene Mx3005p). One μg of total RNA was reverse transcribed using 50 ng random hexamers, 2 mM dNTP mix, 10 u ribonuclease inhibitor, and 200 u MMLV-reverse transcriptase in a final reaction volume of 20 μ l. The primer/probe sets were obtained from Applied Biosystems as the TaqMan Gene Expression Assay kit. The PCR reaction was carried out in a final volume of 20 μl, containing 5 μl of cDNA diluted 1:10, 1× of TaqMan primer/probe mix (20×), and 1× TaqMan® Universal PCR master mix. For each primer/probe tested, the PCR reaction also included a non-reverse transcription negative control to confirm the absence of genomic DNA, a non-template negative control to check for primer-dimer. The amounts of target genes expressed in a sample were normalized to GAPDH. Fold changes between subject groups are measured using the 2^−ΔΔCt^ method, where ΔΔC_T_ = (C_T target_ − C_T normalizer_)_sample_ − (C_T target_ − C_T endogenousgene_)_control_.

### Quantitation of BDNF protein by western blot

Gel electrophoresis and immunolabeling of BDNF were performed by Western blot. Primary neuronal culture cells were homogenized in a Tris buffer containing 5 mM EDTA, 1 mM phenylmethylsulfonyl fluoride, 5 μg/ml each of aprotinin, leupeptin, pepstatin, and 10 μg/ml of 100 mM sodium orthovanadate. Equal volumes of samples containing 10 μ g of protein were electrophoresed on 10% (w/v) polyacrylamide gel. The blots were incubated overnight at 4°C with primary antibody for BDNF (1:1000 dilution; RandD Systems, MN) followed by horseradish-peroxidase-linked secondary anti-mouse or anti-rabbit IgG (1:1000 dilution; GE Healthcare Bio-Sciences, Piscataway, NJ) for 5 h at room temperature. The membranes were stripped and re-probed with β-actin monoclonal primary (1:5000 for 1 h, Sigma Chemical Co., St. Louis, MO) and anti-mouse secondary antibody (1:5000 for 1 h). The bands on the autoradiograms were quantified using the Loats Image Analysis System (Westminister, MD). The optical density (O.D.) of each protein was corrected by the optical density of the corresponding β-actin band. The results are presented as percent of control.

### mRNA expression of BDNF exons

RNA was reverse transcribed using the iScript RT-PCR iQ SYBR Green Supermix (Bio-Rad, Hercules, CA). RT-PCR amplifications were carried out at 50°C for 30 min; 95°C for 15 min followed by 40 cycles of 94°C for 60 s; 57°C for 60 s; 72°C for 60 s and then incubation at 70°C for 10 min, using primers specific to the rat *BDNF* exons (I, II, IV, and VI) as described in Table 1. β-Actin was used as normalizer.

**Table 1:** **Primer sequences for BDNF exons**.

*BDNF* exon I mRNA: CTCAAAGGGAAACGTGTCTCT
TCACGTGCTCAAAAGTGTCAG
*BDNF* exon II mRNA: CTAGCCACCGGGGTGGTGTAA
TCACGTGCTCAAAAGTGTCAG
*BDNF* exon IV mRNA: TGCGAGTATTACCTCCGCCAT
TCACGTGCTCAAAAGTGTCAG
*BDNF* exon VI mRNA: TTGGGGCAGACGAGAAAGCGC
TCACGTGCTCAAAAGTGTCAG

### Methylation-specific RT-PCR for BDNF exon promoter methylation

DNA was isolated from hippocampal neurons, purified and processed for bisulfite modification (Lubin et al., 2008). Methylation-specific PCR primers are provided in Table 2. β-actin DNA was evaluated as control. PCR reactions were performed using the following cycling conditions: 95°C for 3 min, 40 cycles of 95°C for 15 s and 58-60°C for 1 min. Samples were normalized to β-actin and the comparative Ct method was used to calculate differences in gene expression between samples.

**Table 2:** **Methyl-specific Real-time PCR Primers**.

***BDNF* exon I Methylated**
CGGAAAGTATTAGAGGTAGGGTAGC
TACGAACCCTAAATCTCTAAACGAA
***BDNF* exon II Methylated**
TCGTTGTTAAGTTAATTCGGTGTC
AAACTAAAACTAACTCTCCAAACGCT
***BDNF* exon IV Methylated**
GGTAGAGGAGGTATTATATGATAGTTTACG
TAAATAAAAAAAACGACAACGCGAA
***BDNF* exon VI Methylated**
ATTTTTCGGTTTGGAGAAGGAAATC
TCAAAATCCACACAAAACTCTCGAA

### Statistical analysis

Each value is expressed as the mean ± standard deviation (SD). The data were analyzed by SPSS software (Chicago, IL). Statistical differences between each group were evaluated by One-Way analysis of variance (ANOVA) followed by Bonferroni correction. Differences were considered significant at *p* < 0.05.

## Results

### Neuronal death after glutamate treatment and its prevention by lithium

**Figure 1:**
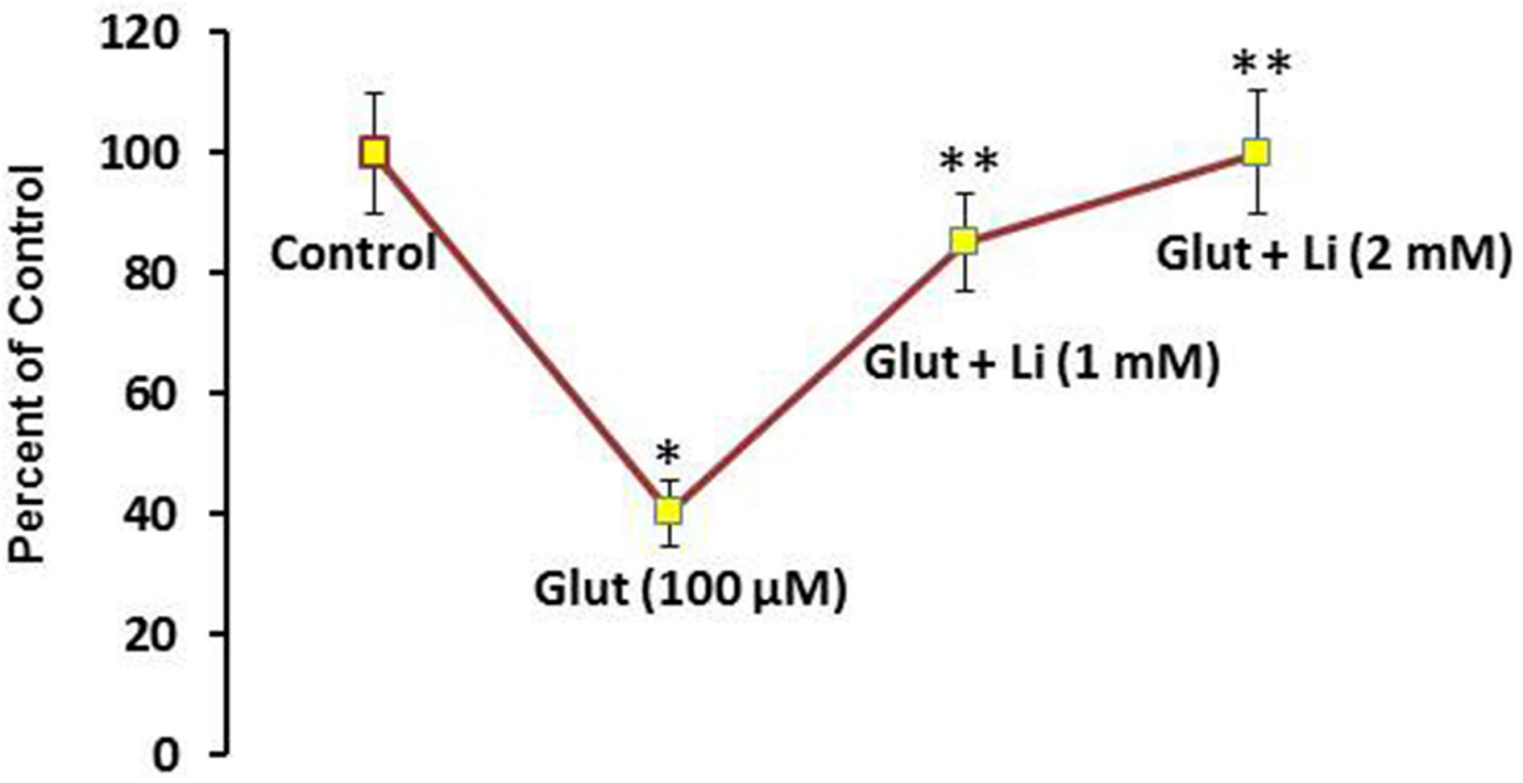
**Pretreatment of lithium prevents neuronal death by glutamate**. Lithium (1 or 2 mM) was added to the medium 36 h prior to glutamate treatment. Glutamate (100 μM) was added 12 h prior to examining neuronal viability. The data are mean ± SD from 3 independent experiments. ^*^P < 0.001 compared with control group and ^**^p < 0.001 compared with glutamate treated group.

To examine whether lithium is neuroprotective, lithium (1 or 2 mM) was added in the culture medium 36 h prior to glutamate (100 μM) addition for 12 h and neuronal viability was assessed. Glutamate reduced the neuronal viability by 60% (Figure 1). It was found that this neuronal death was prevented by lithium in a dose-dependent manner. A dose of 1 mM lithium increased the neuronal viability by 75% whereas 2 mM lithium completely reversed glutamate-induced neuronal death (Figure 1).

### Effect of lithium on dendritic length and number

**Figure 2:**
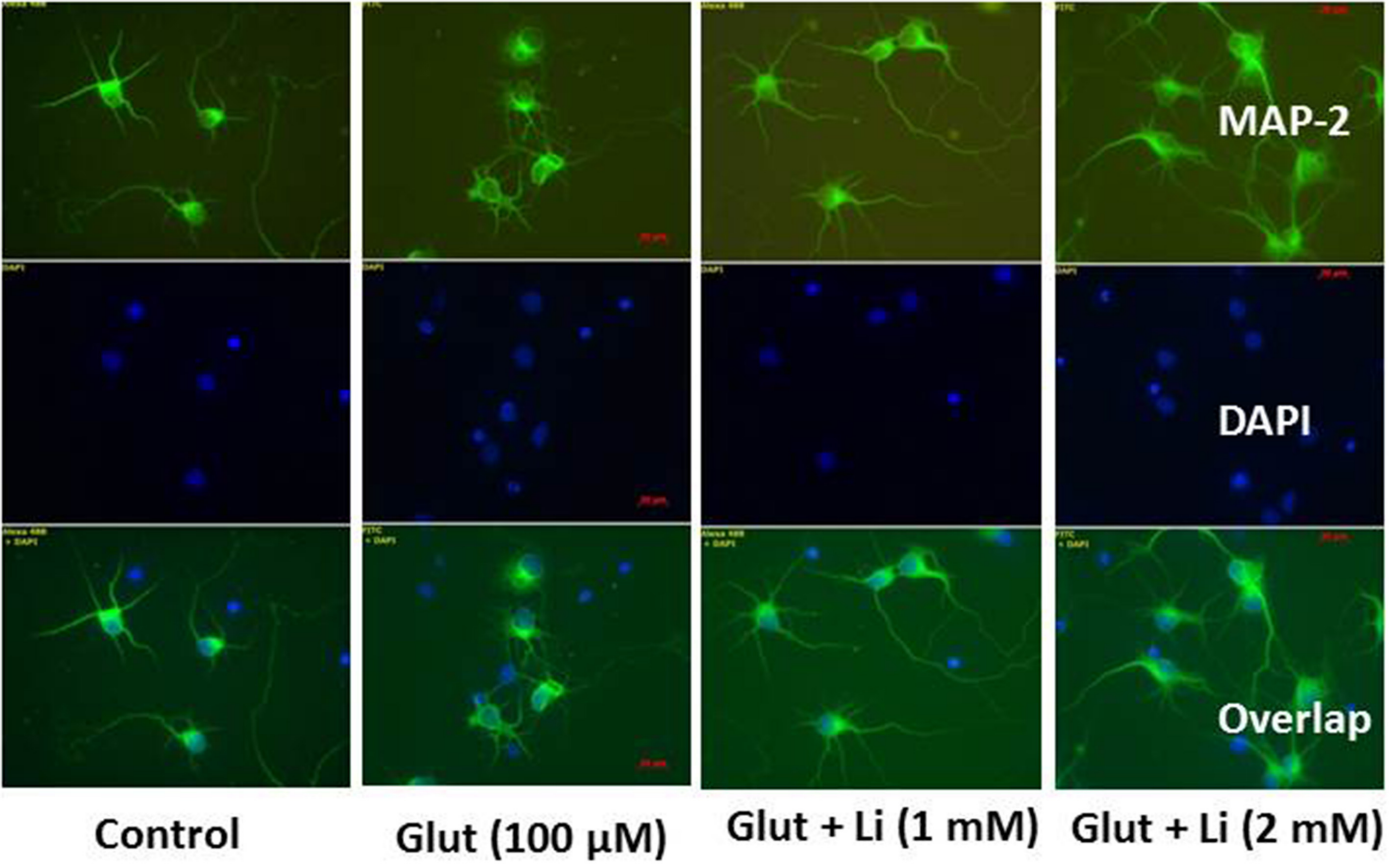
**Neuronal morphology after glutamate (100 μM) and glutamate + lithium (1 or 2 mM) treatment**. Treatment of glutamate and lithium was the same as mentioned in Figure 1.

**Figure 3:**
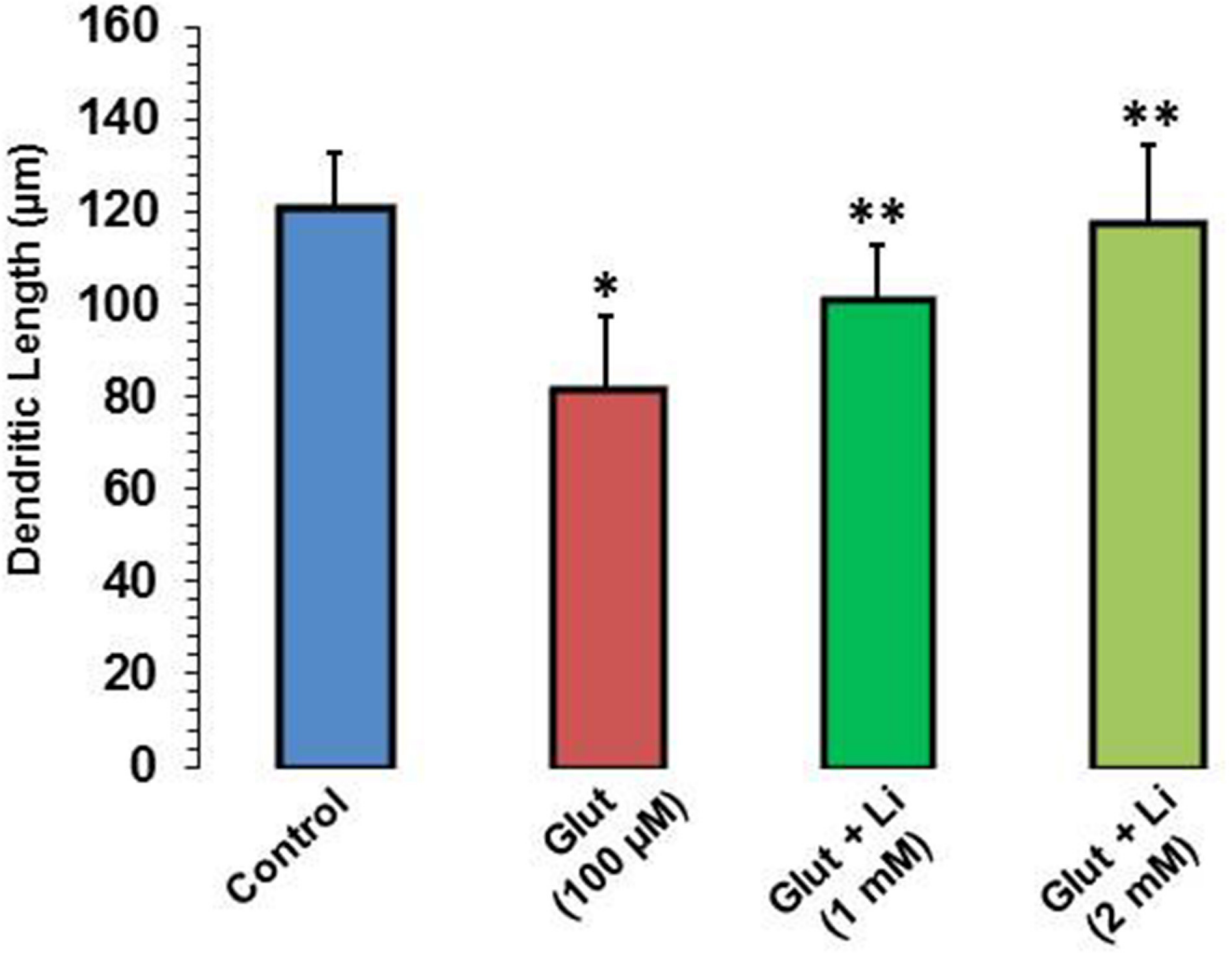
**Dendritic length after glutamate (100 μM) and glutamate + lithium (1 or 2 mM) treatment**. Ten photographs per well were obtained from three wells for each group. Thirty well-stained neurons that made connections to no more than two cells were selected for measurements of their primary dendritic length and dendritic number. ^*^P < 0.001 compared with control group and ^**^p < 0.001 compared with glutamate treated group.

Morphology of hippocampal neurons was studied in the control group (no treatment) and after glulatmate (100 μM for 12 h) or glulatamate + lithium (1 or 2 mM for 48 h) treatment. The morphology of neurons was detected using MAP2 antibody, followed by treatment with DAPI (Figure 2). It was observed that the average dendrite length in the control group was 120.51 ± 12.13 μm. The average dendritic length in the glutamate group was 81.45 ± 16.12. When 1 mM of lithium was added, the average dendrite length increased to 100.64 ± 12.11 μm. Finally, when 2 mM lithium was added, the dendrite length increased to 117.51 ± 17.10 μm (Figure 3).

**Figure 4:**
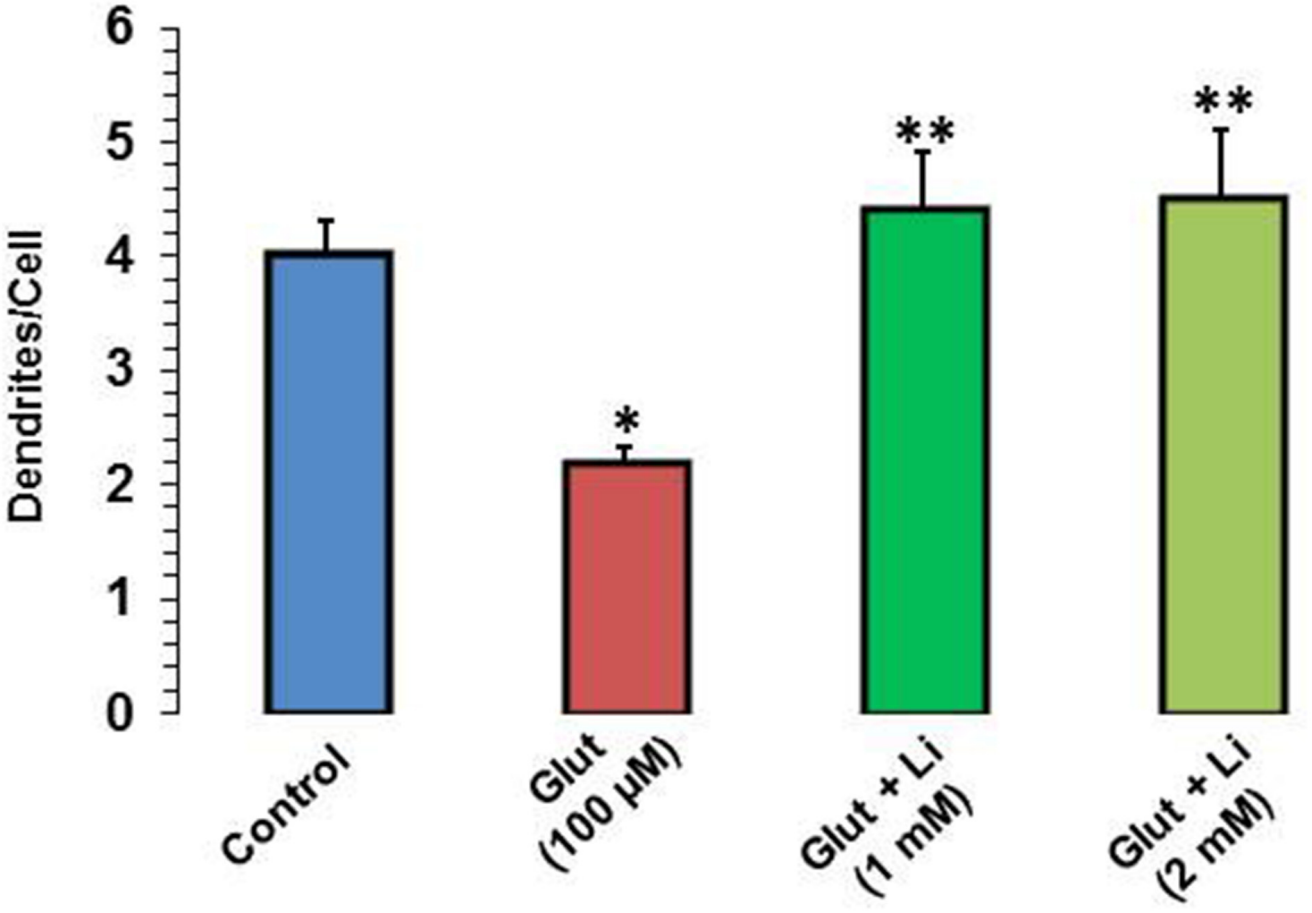
**Dendritic number after glutamate (100 μM) and glutamate + lithium (1 or 2 mM) treatment**. The data are mean ± SD from 3 independent experiments. ^*^P < 0.001 compared with control group and ^**^p < 0.001 compared with glutamate treated group.

When dendrite numbers were compared, it was observed that the average number of dendrites per cell in the control group was 4.0 ± 0.32. Addition of glutamate reduced the dendritic number to 2.2 ± 0.12. The average number of dendrites per cell in glutamate +1 mM lithium group was 4.4 ± 0.51, whereas in glutamate +2 mM lithium group, it was 4.5 ± 0.62 (Figure 4).

### Effect of lithium on mRNA and protein expression of BDNF

**Figure 5:**
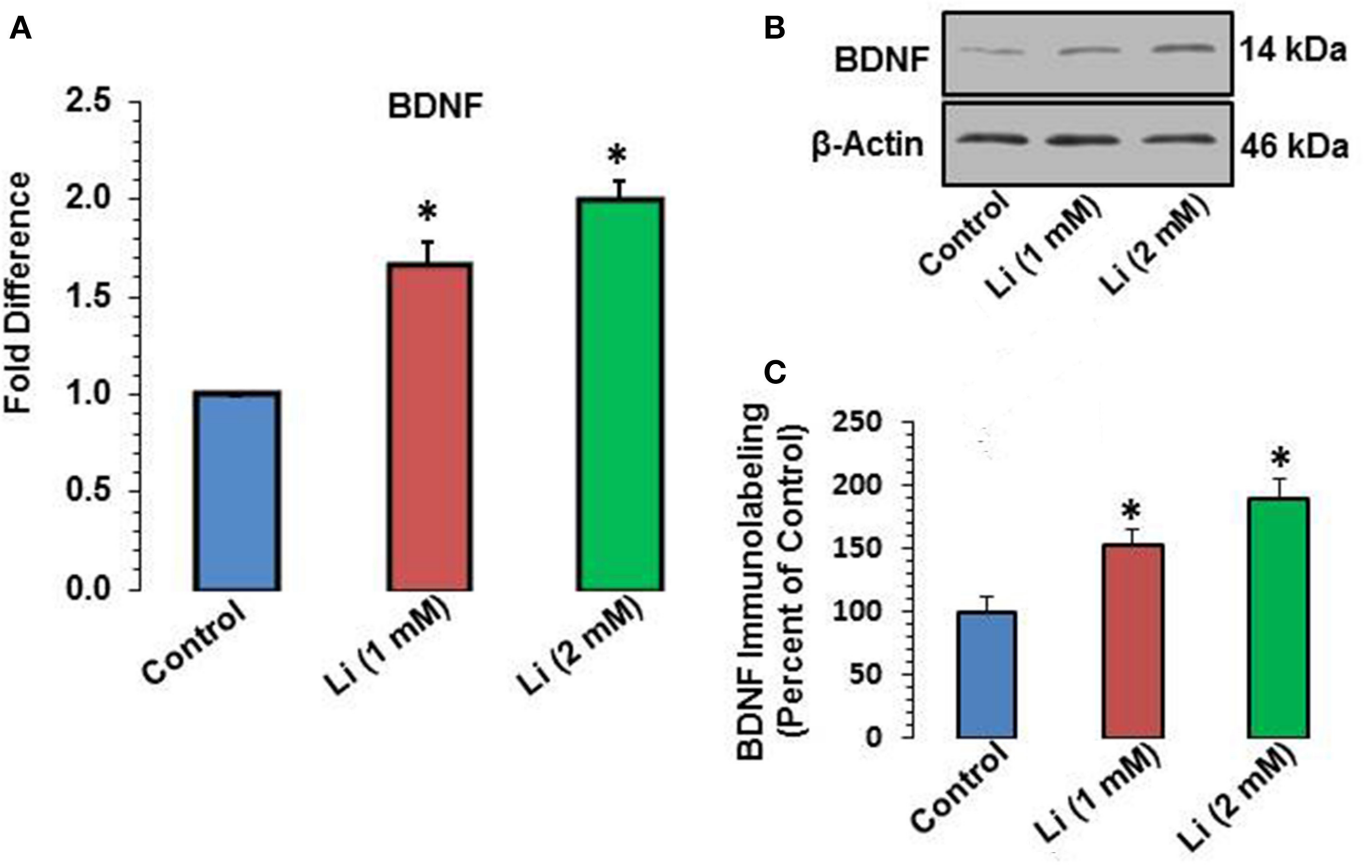
**(A)** BDNF mRNA expression after 48 h of lithium (1 or 2 mM) treatment. ^*^P < 0.001 compared with control group. **(B)** Western blots showing immunolabeling of BDNF and β-actin in hippocampal neurons after 1 or 2 mM lithium treatment. **(C)** Bar diagram showing effect of lithium on protein expression of BDNF. The results are presented as percent of control. The data are mean ± SD from 3 independent experiments. ^*^P < 0.001 compared with saline treated group.

Lithium at 1 mM concentration increased mRNA expression of BDNF. The mRNA expression of BDNF was increased by 67% whereas this effect was further substantiated (100%) at 2 mM lithium (Figure 5A). A Western blot showing expression of BDNF is provided in Figure 5B. When quantitatively assessed, the protein level of BDNF was significantly increased by both 1 and 2 mM concentrations of lithium (Figure 5C). This increase was 53 and 89% for 1 and 2 mM lithium, respectively.

### Effect of lithium on expression of specific BDNF promoter and methylation of promoter exons

**Figure 6:**
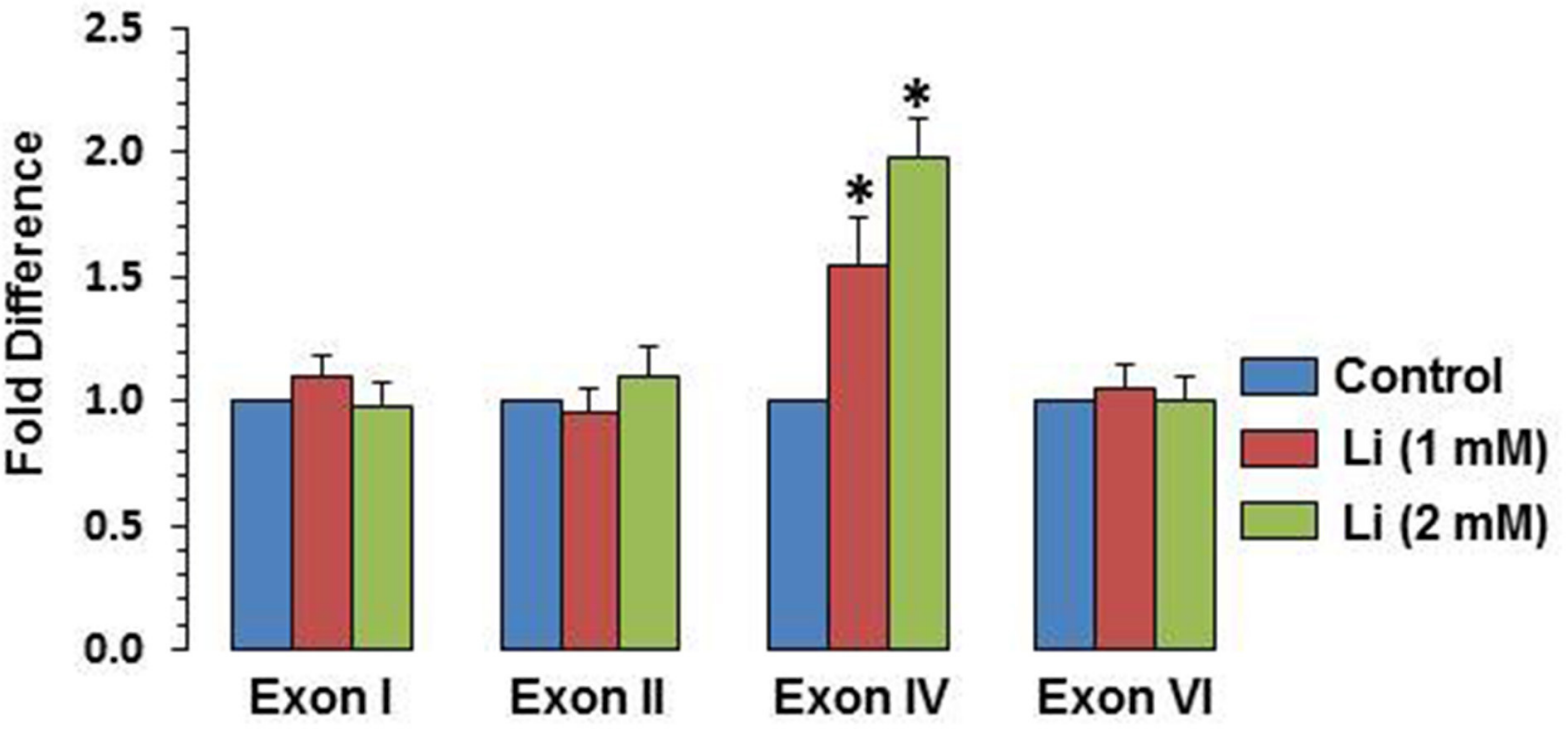
**Effect of lithium (1 or 2 mM) on expression of individual BDNF exons after 48 h after lithium (1 or 2 mM) treatment**. The data are mean ± SD from 3 independent experiments. ^*^P < 0.001 compared with control group.

To examine whether BDNF expression induced by lithium is associated with specific BDNF promoter(s), mRNA level of various exons (I, II, IV, and VI) were examined. It was observed that at 1 mM, lithium increased exon IV expression by 55% whereas at 2 mM, this increase was 98%. There was no significant change in the expression of exons I, II, and VI by lithium (Figure 6).

### DNA methylation of BDNF gene promoters by lithium

**Figure 7:**
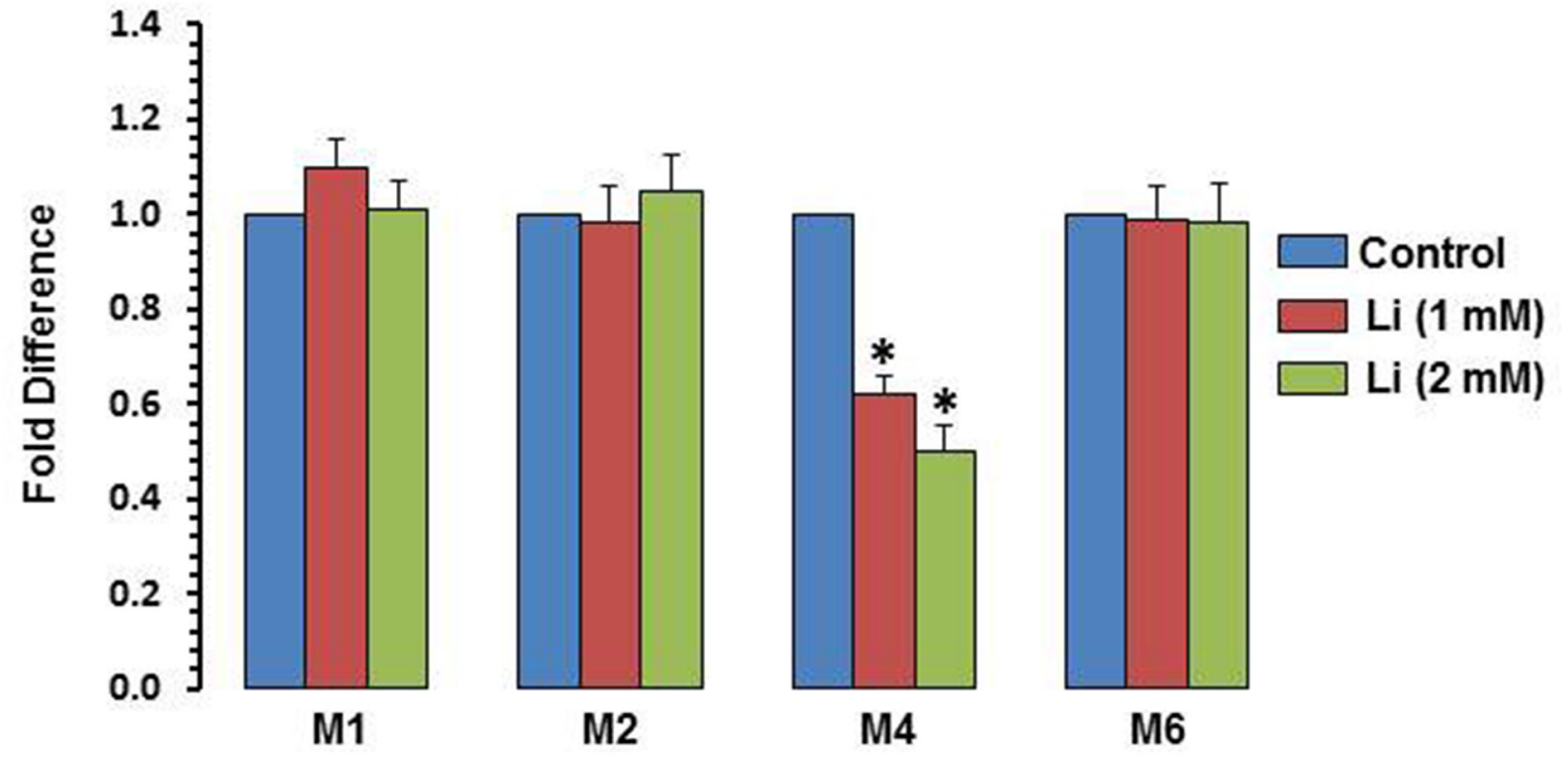
**DNA methylation of BDNF exons after 48 h of lithium (1 or 2 mM) treatment**. The data are mean ± SD from 3 independent experiments. ^*^P < 0.001 compared with control group.

To examine whether the effect of lithium is epigenetically regulated, methylation of exons I, II, IV, and VI was examined. At the dose of 1 mM, lithium decreased methylation of exon IV by 38%, whereas at the dose of 2 mM, this decrease was 50%. Methylation of promoters I, II, and VI was not affected by lithium (Figure 7).

### Effect of lithium on expression of pro- and anti-apoptotic genes

**Figure 8:**
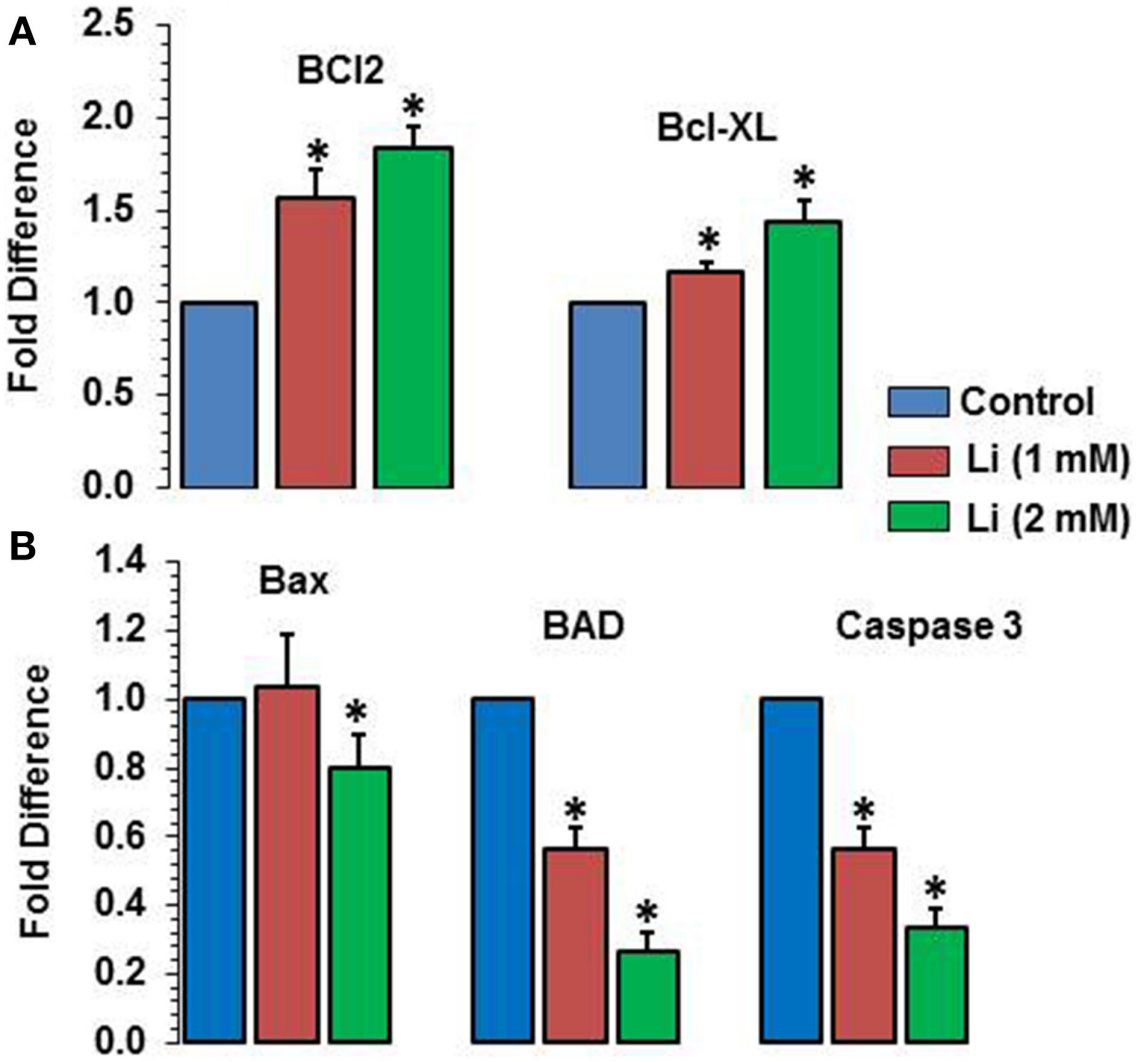
**Effects of 48 h of lithium (1 or 2 mM) on the expression of anti-apoptotic (A) and pro-apoptotic (B) genes**. The data are mean ± SD from 3 independent experiments. ^*^P < 0.001 compared with control group.

As can be seen in Figure 8A, lithium increased the expression of Bcl2 (1 mM of lithium: 1.57 ± 0.15 fold; 2 mM lithium: 1.83 ± 0.12 fold) and Bcl-_XL_ (1 mM of lithium: 1.17 ± 0.06 fold; 2 mM: 1.43 ± 0.12 fold) in a dose dependent manner. Lithium did not show any change in the expression of Bax at 1 mM dose but decreased its expression (0.80 ± 0.10 fold) (Figure 8B). The expression of BAD was decreased by both doses of lithium, the magnitude of change being higher at 2 mM (1 mM lithium: 0.60 ± 0.00 fold; 2 mM of lithium: 0.27 ± 0.06 fold) (Figure 8B). Finally, caspase 3 expression was also decreased by both doses of lithium, higher dose being more effective that the lower dose (1 mM lithium: 0.57 ± 0.06 fold; 2 mM lithium: 0.33 ± 0.06 fold) (Figure 8B).

## Discussion

In the present study, we used two different doses of lithium: 1 mM, which is in the clinical range, and 2 mM, which is slightly higher than the clinical range (reviewed in Young, 2009). Although higher dose has clinical side effects, we wanted to examine whether the side effects are associated with lithium neurotoxicity. Interestingly, this higher dose (2 mM) has been shown to be neuroprotective against amelospheroid-induced toxicity in rat forebrain cholinergic neurons (Hoshi et al., 2003). The neuroprotective action of lithium was tested against glutamate-induced neurotoxicity (Hassel and Dingledine, 2006), which has been shown to be increased in CSF, plasma, and serum (Hoekstra et al., 2006) as well as in brain (Hashimoto et al., 2007) of bipolar patients. Abnormalities in the expression of glutamate receptors (McCullumsmith et al., 2007; Fountoulakis, 2012) and polymorphisms in genes coding for glutamate receptors (Itokawa et al., 2003; Fiorentino et al., 2014; Kandaswamy et al., 2014) have also been reported in bipolar patients. It was found that both doses of lithium were effective in preventing neuronal cell death; higher dose was slightly more effective than the lower dose. From our study, it appears that the higher dose of lithium is neuroprotective and its clinical side effects may be associated with actions other than protecting neurons.

When dendritic morphology was studied, it was found that both doses of lithium increased dendritic length and number. As has been reported, stress plays a major role in mood disorders (Watson et al., 2004) and impaired stress response is associated with hyperglutamatergic state in BD (Zarate et al., 2006). Both stress and hyperglutamatergic state can lead to alterations in spine density and number (Sapolsky, 2000). From our data, it appears that by restoring glutamate-induced changes in neuronal morphology, lithium exerts its neuroprotective action.

BDNF, a molecule implicated in neuronal survival and synaptic plasticity, has been shown to be less expressed in the brain of bipolar patients (Kapczinski et al., 2008). Also, in hippocampus and amygdala of rats showing manic behavior, the expression of BDNF is decreased (Jornada et al., 2010). Under both conditions, lithium normalizes the decreased level of BDNF (Jornada et al., 2010; Wu et al., 2014). As with these studies, the present study also demonstrates that lithium is highly effective in increasing the level of BDNF in hippocampal neurons. To further examine the mechanism of lithium-induced higher expression of BDNF, various BDNF transcripts were examined. The *BDNF* gene in rats consists of nine exons; out of which each exon I, II, IV and VI encodes a 5′-UTR of the BDNF transcript (Aid et al., 2007). When examined, it was found that both 1 and 2 mM doses of lithium increased the expression of selective exon IV. On the other hand, the expression of other exons (I, II, and VI) were not affected. The present study thus suggests that exon IV-containing BDNF transcripts are responsible for total BDNF expression by lithium.

Gene expression in the nervous system can be modulated by various mechanisms. One of the most prominent mechanisms is through epigenetic modifications. In the present study, it was examined whether increased expression of BDNF gene by lithium was modulated via DNA methylation of BDNF exons. Using methylation-specific primers, it was observed that lithium significantly decreased the methylation of the same specific promoter, i.e., promoter IV, which was found to be elevated in hippocampal neurons treated with lithium. The methylation of other promoters such as I, II, and IV was not altered. These results demonstrate that increased expression of BDNF gene is associated with hypomethylation mediated by lithium. Recently, D'Addario et al. (2012) reported that BDNF expression is downregulated in bipolar patients, which was associated with hypermethylation of the BDNF promoter region. These investigators did not study which specific promoter region of BDNF was altered; nevertheless, from the present study, it appears that lithium's clinical efficacy could be associated with alteration in the methylation of the exon IV BDNF transcript.

The present study also shows that lithium dose dependently increased the expression of antiapoptotic genes Bcl2 and Bcl_XL_. On the other hand, lithium decreased expression of pro-apoptotic genes Bad, Bax, and caspase 3. Both the doses were effective in lowering Bad and caspase 3; however, the lower dose was ineffective on Bax. Thus, neuroprotective mechanisms of lithium could also be attributed to its action on apoptotic regulatory genes. Interestingly, a recent study shows that bipolar patients who respond to lithium show increased ratio of anti- to pro-apoptotic genes in blood cells, whereas patients who do not respond to lithium had no significant effect (Lowthert et al., 2012). Thus, it is quite possible that effects on pro- and anti-apoptotic genes may be a prerequisite for lithium action in clinical response to BD.

The mechanism by which lithium modulates the transcription of pro- and anti-apoptotic genes is not clear; however, there may be several possibilities. One could be that BDNF-induced activation of ERK signaling may lead to increased expression of these genes. For example, BCL2 gene contains binding sites for cyclicAMP response element binding protein, a transcription factor that is activated by ERK1/2. Interestingly, lithium has earlier been shown to upregulate ERK expression in cultured cells and in frontal cortex and hippocampus of rat (Chen and Manji, 2006). Lithium also upregulates the expression of BAG-1, a gene that binds to and increases the activity of BCL2 (Zhou et al., 2005). Thus, the effect of lithium on BCL2 could be associated with increased expression of BAG-1. The other possibility could be of increased RNA polymerase activity and thereby increased gene expression by lithium. Further studies will be needed to examine such mechanisms.

Overall, this study provides several mechanisms by which lithium may exert its neuroprotective action. Most prominently, its action on DNA methylation of promoter IV of BDNF and on apoptotic regulatory proteins appears to be directly related to its neuroprotective action. Another point to be noted is that although done in *in-vitro*, nevertheless, our study clearly shows that even higher dose was effective in protecting neurons. Paradoxically, the higher doses >2 mM have been shown to have several side effects; however, they do not appear to be associated with neurotoxicity as 2 mM was effective in reversing glutamate-induced neural viability and protecting neurons. These studies must take into account the limitations presented by an *in-vitro* study and needs to be validated in *in-vivo* to fully evaluate lithium's neuroprotective activity in the human population.

### Conflict of interest statement

The authors declare that the research was conducted in the absence of any commercial or financial relationships that could be construed as a potential conflict of interest.
